# Maxillary sinus augmentation using chairside bone marrow aspirate concentrates for implant site development: a systematic review of histomorphometric studies

**DOI:** 10.1186/s40729-018-0137-3

**Published:** 2018-09-03

**Authors:** Miriam Ting, Philip Afshar, Arik Adhami, Stanton M. Braid, Jon B. Suzuki

**Affiliations:** 1 Private practice in Periodontology, and Think Dental Learning Institute, Paoli, PA 19301 USA; 2 Temple University Kornberg School of Dentistry, 3223 North Broad Street, Philadelphia, PA 19140 USA; 3 Division of Oral and Maxillofacial Surgery, Department of Oral Medicine, Pathology and Surgery, Temple University Kornberg School of Dentistry, 3223 North Broad Street, Philadelphia, PA 19140 USA; 4 Department of Microbiology and Immunology, Temple University (Medicine), 3223 North Broad Street, Philadelphia, PA 19140 USA; 5 Department of Periodontology and Oral Implantology, Temple University (Dentistry), 3223 North Broad Street, Philadelphia, PA 19140 USA

**Keywords:** Bone marrow aspirate concentrates, Histomorphometric, Bone grafting

## Abstract

Maxillary sinus pneumatization following dental tooth extractions and maxillary alveolar bone resorption frequently leaves inadequate bone levels for implant placement. The objectives of this systematic review are to evaluate the effects of bone marrow aspirate concentrates (BMACs) used in maxillary sinus augmentation for implant site development.

A systematic search was conducted using PubMed, EMBASE, Web of Science, Cochrane Library, and Google Scholar for studies which histomorphometrically evaluated the efficacy of BMACs and BMAC-enriched biomaterials in sinus floor elevation. Six studies were selected, and the risk of bias was evaluated.

Reported ranges of vital mineralized tissue for the BMAC groups for the selected studies were 34.63–55.15% compared to 27.30% for control groups. For vital mineralized bone, these studies reported variable statistical significance. At 3–4 months, new bone formation for BMAC groups with controls using no BMAC was 7.4–12.6% and for the control groups was 9.45–14.3%. At 6 months, new bone formation for BMAC groups was 13.5–14.12% and for control groups was 10.41–13.9%. For new bone formation, these studies reported no significant difference between test and control and between 3 and 6 months histologic evaluation.

Within the limits of this systematic review, the chairside method to harvest BMAC produced similar implant survival and new bone formation compared to the laboratory FICOLL group, without the additional cost and time of laboratory cell isolation techniques. The iliac crest or tibia origins, single or double centrifugation, for BMAC do not appear to be a factor for implant survival or bone formation. Although some favorable outcomes were reported, the increase in new bone formation using chairside-harvested BMAC compared to control is not predictably more significant across studies.

Clinically, new bone formation in the maxillary sinus is not always contingent on the presence of BMAC. The novelty of this method requires more future studies.

## Review

Maxillary sinus augmentation is indicated when there is an inadequate vertical alveolar bone height to effectively support surgically placed dental implants. The sinus elevation procedure requires grafting bone material onto the sinus floor to regenerate sufficient vertical alveolar bone height [1, 2]. The ideal bone grafting material should be biocompatible, possess no risk of disease transmission, promote bone regeneration, and have mechanical stability throughout the healing period [3]. Autologous bone is the “gold standard” of bone grafting materials in maxillary sinus lifts due to its osteoconductive, osteoinductive, and osteogenic potential [4, 5]. The harvesting of autologous bone is highly invasive and time-consuming and has variable outcomes for donor and recipient sites [5, 6]. Current biomaterials like xenografts, homologous grafts, and synthetic grafts circumvent the risks of autologous grafts but lack cellularity [7]. Bone marrow aspirate concentrates (BMACs) alone and biomaterials enriched with BMACs were proposed to have the potential to increase the success of sinus floor elevation surgeries, rather than biomaterials alone [8]. Autologous bone marrow is a known source of undifferentiated mesenchymal cells that can differentiate into osteoblasts [9] and produce vascular endothelial growth factors [10].

The technique involving bone marrow-derived mononuclear cell (MNC) isolation by synthetic polysaccharide (FICOLL) is considered an optimum approach for harvesting of MNCs [11]. It is primarily used in orthopedics and requires good manufacturing practice laboratory techniques. Thus, the closed bone marrow aspirate concentrate (BMAC) system which can be done chairside has become an accepted means to harvest MNCs [12].

The objective of this systematic review is to evaluate the histomorphometric outcomes of BMAC harvested chairside on regeneration of bone in maxillary sinuses grafted for implant site development.

## Materials and methods

### Focus question

What are the histomorphometric outcomes of sinus augmentation with bone marrow aspirate concentrates obtained chairside?

### Literature search

PubMed, Web of Science, Cochrane Library, and Google Scholar were searched up to January 2017. Google scholar was searched for gray literature. The following keywords were used: “bone marrow aspirate concentrates,” “stem cells,” “histomorphometric,” and “bone grafting.” The reference list of the selected articles was further hand searched for any articles not included in the initial search.

### Inclusion and exclusion criteria

- Clinical studies involving only intraoral bone grafting applications of BMAC in the maxillary sinus of humans were included
- Clinical studies including histomorphometric evaluation were included
- Only chairside BMAC methods of harvesting BMAC are included
- Animal studies, in vitro studies, and case reports are excluded

### Screening and data extraction

The “title and abstract” were independently screened by two reviewers (AA, PA); articles were excluded if they obviously did not meet the inclusion criteria. The full text was independently analyzed by three reviewers (AA, PA, MT). A previously pilot-tested data extraction sheet was used by two reviewers (PA, MT) to independently extract data. Any disagreements were resolved through discussion with a fourth reviewer (SMB).

### Assessment of risk of bias

The risk of bias tool [13] by the Office of Health Assessment and Translation (OHAT) was used to assess the risk of bias (Table 1). Two reviewers (MT and PA) independently scored the risk of bias for the selected studies, and disagreements were resolved through discussion with another reviewer (SMB).

**Table 1:** OHAT risk of bias assessment

	Study					
	de Oliveira et al. [12]	Pasquali et al. [7]	Payer et al. [2]	Sauerbier et al. [11]	Sauerbier et al. [14]	Wildburger et al. [15]
Selection bias						
Was administered dose or exposure level adequately randomized?	++	++	++	−−	++	++
Was allocation to study groups adequately concealed?	NR	NR	NR	−−	+	NR
Performance bias						
Were the research personnel and human subjects blinded to the study group during the study?	−−	−−	−−	−−	+	−−
Detection bias						
Can we be confident in the exposure characterization?	−	−	−	−	−	−
Can we be confident in the outcome assessment?	++	++	++	++	++	++
Attrition bias						
Were outcome data complete without attrition or exclusion from analysis?	++	++	+	++	−	−
Reporting bias						
Were all measured outcomes reported?	++	++	++	++	−	−

*++* definitely low risk of bias, *+* probably low risk of bias, *−/NR* probably high risk of bias, *−−* definitely high risk of bias, *NR* not reported

## Results

**Fig. 1:**
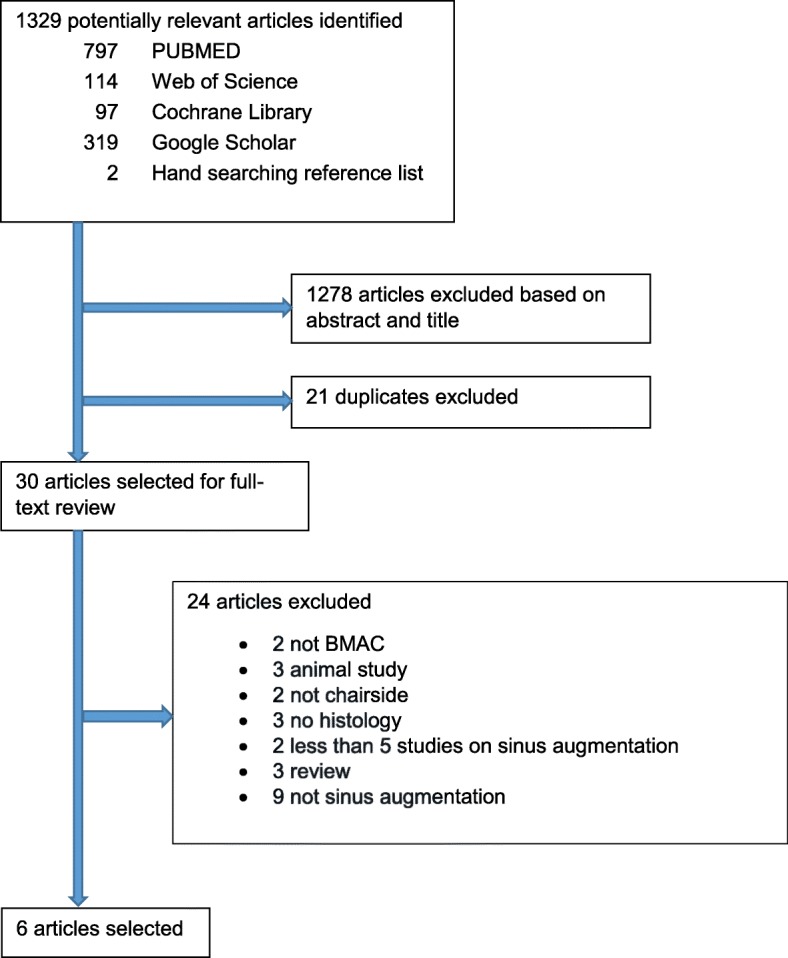
Search strategy for BMAC

The search generated 797 reviews in PubMed, 114 in Web of Science, 97 in Cochrane Library, and 319 in Google Scholar (Fig. 1). The following were selected after the title and abstract screening: 18 were selected from PubMed, 23 from Web of Science, 6 from Cochrane Library, 2 from Google Scholar, and 2 from hand searching the reference list of the selected article. After the duplicates were removed, 30 articles remained for full-text analysis. Twenty-four were eliminated after full-text analysis, and six articles remained for data extraction (Table 2). The risk of bias of the selected studies scored mostly “definitely low risk of bias” or “probably high risk of bias” (Table 1).

**Table 2:** Characteristics of selected studies

Study	Treatment groups	No. of patients (age range)	No. of maxillary sinuses evaluated	Donor site for BMAC	Clinical findings/implant survival	Timing of biopsy	Histologic outcomes	
de Oliveira et al. [12]	SCG (BMAC obtained by single centrifugation and bone graft) DCG (BMAC obtained by double centrifugation and bone graft) Control (xenogenous bone graft alone)	15 (mean age 55.4 years)	21	Posterior iliac crest	- 68 implants were placed in the previously grafted sites, and 100% osseointegrated - Loading was applied after 6 months	6 months	Vital mineralized tissue: - SCG 38.44% - DCG 34.63% - Control 27.30% - Not statistically different Non-vital mineralized tissue: - SCG 13.70% - DCG 19.63% - Control 22.79% - Not statistically different	Non-mineralized tissue: - SCG 47.87% - DCG 45.73% - Control 49.90% - Not statistically different
Pasquali et al. [7]	Test (BMAC and xenogenous bone graft) Control (xenogenous bone graft alone)	8 (mean age 55.4 years)	16	Superior posterior iliac crest	- At least two implants were placed in each previously grafted sites, and all implants osseointegrated - Loading was applied after a 6-month healing period	6 months	Vital mineralized tissue: - Test 55.15% - Control 27.30% - Statistically significant Non-vital mineralized tissue: - Test 6.32% - Control 22.79% - Statistically significant	Non-mineralized tissue: - Test 38.53% - Control 49.90% - Not statistically significant
Payer et al. [2]	Test (porous bovine bone mineral together with tibial bone marrow aspirate) Control (bovine bone graft without any additive)	6 (mean age 58.2 years)	12	Tibia	- 44 implants were stable and osseointegrated at radiographic and Periotest evaluation	3 months 6 months	Newly formed bone: 3 months -Test 10.36% - Control 9.45% - Not statistically significant 6 months - Test 14.12% - Control 10.41% - Not statistically significant 3 and 6 months: - Not statistically significant	Bone-to-bone substitute contact: 3 months -Test 16.40% - Control 15.06% - Not statistically significant 6 months - Test 20.26% - Control 17.89% - Not statistically significant 3 and 6 months: - Not statistically significant
Sauerbier et al. [11]	Open bone marrow-derived mononuclear cell isolation by synthetic poylsaccharide (FICOLL) method with bovine bone mineral (BBM) Closed bone marrow aspirate concentrate (BMAC) system with BBM	FICOLL group 4 (mean age 59.5 years) BMAC group 7 (mean age 55 years)	FICOLL group 6 BMAC group 12	Superior posterior iliac spine	- 50 implants were placed (17 FICOLL group and 33 BMAC group) - Implant survival was evaluated after 1 year - No implant out of 17 was lost in the FICOLL group, before prosthetic loading - 1 implant out of 33 failed in the BMAC group, before prosthetic loading - No implant was lost after loading - All 49 osseointegrated implants were loaded and in function	3 months	New bone formation: - FICOLL group 15.5%, - BMAC group 19.9%, - Not statistically significant Value of biomaterial: - FICOLL group 19.7% - BMAC group 31.9% Value of marrow space: - FICOLL group 64.8% - BMAC group 47.4% - Marrow space is significantly less for BMAC	
Sauerbier et al. [14]	Test: bovine bone mineral (BMM) and BMAC Control 70% BBM and autogenous bone 30% harvested from the retromolar area	25 test 11 control (mean age 56.6 years)	34 test 11 control	Superior posterior iliac spine	Radiologic gain and augmented bone height: - Test 1.74% - Control 1.33% - Statistically significant	3–4 months	New bone formation: - Test 12.6% - Control 14.3% - Not statistically significant	
Wildburger et al. [15]	Test: bovine bone mineral (BBM) mixed with a concentrate harvested from the posterior iliac crest Control: BBM alone	7 (mean age 58 years)	14	Superior posterior iliac crest	- 52 implants were placed - Average dental implant healing time was 4 months - Prosthetic treatment was achieved in all patients	3 months 6 months	New bone formation: 3 months - Test 7.4% - Control 11.8% - Not statistically significant 6 months - Test 13.5% - Control 13.9% - Not statistically significant	Fraction of bovine bone material 3 months - Test 42.6% - Control 34.9% 6 months - Test 36.2%, - Control 39.5%

*BMAC* bone marrow aspirate concentrate

Most of the selected six studies reported that new bone formation and other histomorphometric outcomes were not statistically different between control and test sites. Only one study reported a significant increase in new bone formation between BMAC + bovine bone graft test sites compared to bovine bone graft controls. Another study reported histologic outcomes of BMAC-grafted sites to produce as much new bone as the traditional laboratory-based method. Most studies also reported that all implants placed in both test and control sites were osseointegrated, successfully loaded, and in function.

The reported ranges of vital mineralized tissue for the included studies for the BMAC groups were 34.63–55.15% compared to 27.30% for control groups [7, 12]. For vital mineralized bone, these studies reported variable statistical significance. At 3–4 months, new bone formation for BMAC groups with controls [2, 14, 15] using no BMAC was 7.4–12.6% and for the control groups was 9.45–14.3%. At 6 months, new bone formation for BMAC groups [2, 15] was 13.5–14.12% and for control groups was 10.41–13.9%. For new bone formation, most studies reported no significant difference between the test and control and between the 3 and 6 months histologic evaluation.

## Discussion

Mesenchymal stem cells (MSCs) in BMAC have the potential to renew, experience clonal expansion, and differentiate into musculoskeletal tissues [16]. MSCs are also known to have an immunoregulatory role and may enhance the normal healing response and angiogenesis [10]. BMAC has been used in bone, cartilage, and tendon injuries with encouraging results [16]. BMAC is a minimally invasive procedure, avoiding the risks of an open bone graft procedure, but still requires the same care and consideration for asepsis.

The published clinical and histomorphometric studies [2, 7, 11, 12, 14, 15] were generally seeking the same clinical outcome: implants surgically placed in bone regenerated by selected tissue engineering approaches. Generally, BMAC derived by the iliac crest or tibia is mixed with bovine bone (test group) and compared with bovine bone alone (control group) after placement into the maxillary sinus.

Sauerbier et al. [12] compared preparation techniques for mesenchymal stem cells. BMAC + bovine bone was compared with FICOLL–Hypaque centrifugation preparation of BMAC after placement into maxillary sinuses. New bone (19.9%) from the test group and new bone from the control group (15.5%) were not statistically significant. FICOLL preparation in this study included centrifugation of BMAC at 2400 rpm for 25 min. Cell preparations were rinsed 2× in phosphate-buffered saline (PBS). Trypan blue dye exclusion of small aliquots of the final product were evaluated microscopically, but percentage of viable cells was not presented. This study indicated that BMAC + bovine bone grafts were equal to BMAC FICOLL preparation, despite the 50-min laboratory time taken to purify cells. Histologic diagnosis of this paper was highly sophisticated and used bar graphs with standard deviation (SD) to illustrate differences between groups. New bone was stained by Azar II–Pararosanilin and new bone formation assessed in both native bone and bovine bone samples. Confidence limits (Cl) of greater than 90% were reported for the differences in new bone with BMAC vs FICOLL preparations. Since FICOLL cell preparation was the standard method to purify bone regeneration cells before chairside BMAC was developed, it was important to recognize the effectiveness of chairside BMAC compared to the existing standards of FICOLL cell preparation.

Sauerbier et al. [14] further compared BMAC + bovine bone grafts (test group) with alveolar bone, autologous + bovine bone grafts (control group) for maxillary sinus site preparation. New bone (31.3%) for the test group compared with new bone (19.3%) for the control group statistically indicated equivalence in histomorphometric outcome. Histologic images showing impressive new bone formation were presented. BMAC + bovine bone was equivalent to alveolar bone + bovine bone 3–4 months postsurgically. Histologic diagnosis employed standardized formalin-fixed preparations, serially dehydrated with alcohol in increasing concentrations. In addition, 100-μm sections were analyzed and data reported in bar graphs of BMAC + bovine bone vs alveolar bone alone. Proof of multipotency for three cell types demonstrated that chondrogenic, adipogenic, and osteogenic activities were present.

Payer et al. [2] compared BMAC derived from tibia + bovine bone (test group) to bovine bone alone (control) for maxillary sinus augmentation. Comparisons at 3 and 6 months were recorded with Periotest and radiographs. And similar results were reported. BMAC derived from the tibia resulted in no benefit for bone regeneration but was suitable as a donor for bone grafts. Due to “high variation of data” and “low number of patients” in this study, these outcomes need further evaluation. Histologic analyses for this study employed 30-μm-thick histologic samples. Images for new bone formation were evaluated for percentage bone contact with software programs. Flow cytometry diagnosed phenotype of bone marrow stromal cells demonstrating the same immunophenotype for cells.

Wildburger et al. [15] compared mesenchymal bone cells + bovine bone (test group) with bovine bone alone (control) in seven patients requiring bilateral maxillary sinus lift site preparation surgeries. Three months postsurgically, bone biopsies were taken with implant drills. New bone (13.5%) for the test group was compared with new bone (13.9%) for the control group. There was no statistical difference in the test vs control bone graft groups in the maxillary sinus. Histologic diagnosis evaluation for this study employed 300–400-μm-thick specimens. Azar II + Pararosanilin stains were used to evaluate new bone formation (red stain) vs bovine bone (green stain). Fluorescence-activated cell sorting (FACS) analyses of cell cultures confirmed cell differentiation.

Pasquali et al. [7], in eight patients compared BMAC + bovine bone graft (test group) with bovine bone graft alone (control group). New bone (55.15%) was reported in the test group compared with new bone (27.3%) in the control group based on histomorphometric analyses. This reported observation indicating statistically more new bone regeneration in the BMAC + bovine bone graft group compared with control bovine bone graft group needs further investigation. Histologic diagnosis for this paper used Masson’s Trichrome stain. Histological assessment was divided into square-millimeter segments and new bone; vital vs non-vital vs non-mineralized bone was assessed by two independent examiners. Microscopically, square millimeters of new bone was reported as percentage of area. BMAC samples were consistently (eight patients) better than controls (bovine bone graft alone). However, subtle clinical or laboratory preparation techniques not described in the details of the “Materials and methods” section may account for these improved results.

Finally, de Oliveira et al. [12] compared BMAC laboratory preparation techniques involving one or two centrifugations. The first test group was BMAC with single centrifugation + bovine bone graft. The second test group was BMAC with double centrifugation + bovine bone graft, perhaps resulting in greater purity. The control group was bovine bone graft alone. Six months postsurgically, biopsies were performed and the specimens submitted for histomorphometric analyses. New bone (38.4%) resulted with the single BMAC centrifugation. New bone (34.63%) resulted with the double BMAC centrifugation. The control group had 27.3% new bone. Therefore, although the single BMAC centrifugation resulted in the best histomorphometric outcome, it was not statistically significant. Histologic diagnosis employed biopsies decalcified by 10% EDTA for 36 h. Seven-micron sections were evaluated histologically for non-vital bone, vital bone, and non-mineralized bone. No histologic difference between single centrifugation of BMAC (38.44%) vs double centrifugation of BMAC (34.63%) was reported. Controls (bovine bone alone) resulted in 27.3% new bone formation.

It appears that BMAC offers no statistically significant advantage for regeneration of bone in the maxillary sinus for site preparation of dental implants. BMAC + bovine bone graft results in similar regeneration outcome measures histologically as alveolar bone alone at 3–4 months. Measured histomorphometrically MSCs treated by FICOLL–Hypaque centrifugation to consolidate osteogenic and osteoinductive cells afford no statistically significant advantage for bone regeneration compared to BMAC [11].

The origin of BMAC whether from the iliac crest or proximal tibia appears not to impact histomorphometric improvements in bone regeneration cells, although data are quite limited in BMAC origins [2].

The method of laboratory preparation of BMAC whether single centrifugation or double centrifugation does not improve histomorphometric enhancement of bone regeneration potential inclusion of cells or new bone [12]. Bovine bone graft materials alone appear to consistently result in bone regeneration in the maxillary sinus in preparation for dental implant site preparation enhancement of bone [14].

Further histomorphometric and clinical studies are needed with improved consistency of clinical methods and laboratory preparation of biopsy materials. The samples are evaluated microscopically. An evaluation of the samples in the published studies may produce different interpretations due to variance of thickness of specimens, 7-μm sections in the de Oliveiro paper [12], 30-μm sections for the Payer paper [2], and 300–400-μm sections for the Wildburger paper [15]. Standardized histologic protocols may reduce the imaging interpretation inconsistencies.

Also, stains for bone specimens are not congruent between studies. Azar II + Pararosanilin stains for bone formation determination was used by Wildburger et al. [15] and both Sauerbier et al. papers [11, 14]. Masson’s Trichrome stain, a more conventional histological laboratory stain, was used to assess bone formation in the Pasquali et al. paper [7].

The maxillary sinus lateral wall surgical approaches for site preparation and bone regeneration were fairly standardized in all the papers evaluated. The time of 6 months wound healing is a consistent pattern of assessment and should be continued in future studies [2, 7, 12, 15].

Although the variations of the materials and methods for BMAC preparation were discussed, this review was not aimed to compare materials and methods for BMAC preparation, but rather the end clinical result for new bone formation and implant survival. Although implant survival and new bone formation were not the only parameters to consider when evaluating sinus augmentation, these parameters were the only ones consistently evaluated and reported by the existing literature to date; thus, these parameters were the ones emphasized in this systematic review. However, it is important to realize that BMAC may be of significance for other aspects of wound healing during sinus augmentation like immediate post-operative pain or soft tissue healing. And parameters should be evaluated in future studies of BMAC use in maxillary sinus augmentation.

Collectively, the papers selected for this systematic review were well written and peer-reviewed by clinicians in private practice and academic professors. However, risk of bias cannot be avoided. Sauerbier et al. [14] used selective bar graphs for histological diagnoses. Bar graphs have the potential for bias when the *x* and *y* axes are skewed to favorable results. Payer et al. [2] compared bovine with tibia bone marrow aspirate plus bovine bone. Their data show high variation of data and low numbers of patients. The result of no benefit to tibia bone marrow aspirate may be biased by the low numbers of patients. Histologically, Payer et al. [2] used 30-μm sections showing images of new bone reported as percentage of bone contact. The varying thickness of micron sections in histological preparation could influence bone contact data and percentage of new bone formation, even though the flow cytometry used to diagnose phenotypes of bone marrow stromal cells is very innovative and highly accurate. Furthermore, to reduce risk of bias, dual examiners to standardize histologic interpretation should be utilized in clinical studies of this type.

In addition, the parameters to evaluate new bone formation were variable. New bone formation and percentage of vital bone are different methods to measure bone formation, and the data from different methods could not be combined or analyzed together. Furthermore, the BMAC evaluated in test groups were prepared differently and were harvested from different sources (tibia or iliac). The control groups with no BMAC were also slightly different and included different graft materials. Thus, the heterogeneity of the data prevents any meaningful data analysis.

## Conclusions

Within the limits of this systematic review, the chairside method to harvest BMAC is a viable option for maxillary sinus augmentation for implant site development. The implant survival of the BMAC group was similar to the laboratory FICOLL concentration of BMAC group, without the additional cost and time of laboratory cell isolation techniques. Single or double centrifugation of BMAC does not appear to be a factor for new bone formation. The iliac crest or tibia origins for BMAC do not appear to be a factor for implant survival or bone formation. The use of BMAC can induce new bone formation comparable to control sites with no BMAC. However, the increase in new bone formation using chairside-harvested BMAC compared to controls with no BMAC is not predictably more significant across studies. In a clinical situation, new bone formation and regeneration in the maxillary sinus is not necessarily contingent on the presence of BMAC. And future studies should be directed at standardizing cell preparation methods and stains, as well as other parameters involved in wound healing of the maxillary sinus.

## Abbreviations

BMAC: Bone marrow aspirate concentrate
CI: Confidence limits
EDTA: Ethylenediaminetetraacetic acid
FICOLL: Technique involving bone marrow**-**derived mononuclear cell isolation by synthetic polysaccharide
MNCs: Mononuclear cells
MSCs: Mesenchymal stem cells
OHAT: Office of Health Assessment and Translation
SD: Standard deviation
